# Stoichiometric effect on the defect states and optical properties of LiInSe_2_ single crystals

**DOI:** 10.1038/s41598-024-61547-9

**Published:** 2024-10-21

**Authors:** Zhi Zheng, Hui Yu, Menghua Zhu, Zheren Zhang, Zhihui Gao, Meng Xu, Rui Zhang, Yadong Xu

**Affiliations:** 1 State Key Laboratory of Nuclear Power Safety Technology and Equipment, China Nuclear Power Engineering Co., Ltd., Shenzhen, 518172 Guangdong China; 2 State Key Laboratory of Solidification Processing, School of Materials Science and Engineering, Northwestern Polytechnical University, Xi’an, 710072 China; 3 Shaanxi Imdetek Co., Ltd., Xi’an, 712000 China

**Keywords:** LiInSe_2_, Stoichiometric effect, Point defect, Band gap, Physical chemistry, Chemical synthesis, Optical physics, Chemistry, Physics

## Abstract

LiInSe_2_ crystals are promising semiconductor materials for neutron detectors due to the large neutron capture cross-sectional area of the specific isotopes (^6^Li) and high charge transport properties. However, the optoelectronic performance fails to reach the expected level due to the difficulty of controlling the crystal defects. Herein, we modulate the stoichiometric ratio to control the type of defects in single LiInSe_2_ crystals grown by the vertical Bridgman method. The UV‒vis–NIR transmission results indicate that the band gap of the yellow colored Li_1.01_In_1_Se_2_ sample is close to ~ 2.83 eV at room temperature, and this value is consistent with the theoretical band gap of LiInSe_2_ (~ 2.86 eV). Photoluminescence (PL) spectroscopy was used to analyze the defect concentration. The results indicate that the defect types in the yellow Li_1.01_In_1_Se_2_ single crystal are V_Se_^+^ and Li_In_^2−^; these result from the introduction of excess Li and the suppression of the adverse defects in In_Li_^2+^ and V_Li_^−^. These results demonstrate a feasible route for obtaining high-quality yellow ^6^LiInSe_2_ crystals and promote the application of ^6^LiInSe_2_ neutron detectors.

## Introduction

High-sensitivity, large-size neutron detectors play vital roles in industrial and biomedical applications^1–5^. ^3^He-based gaseous, scintillator, and semiconductor-based detectors dominate the commercial market^6^. Compared with gas and scintillators, compact structured semiconductor detectors exhibit high spatial resolution and fast time response due to the high energy resolution and wide linear range of semiconductors; these detectors are suitable for preparing an efficient neutron detector^7,8^.

LiInSe_2_ is a new type of semiconductor with a chalcogenide structure and has a large nonlinear optical coefficient and a suitable transparent wavelength range^9,10^. Importantly, ^6^LiInSe_2_ crystals display a remarkable ability to reflect ionizing radiation via the nuclear reaction of ^6^Li(n,*α*)^3^H and direct charge transport^11,12^. A high density of ^6^Li cations in the ^6^LiInSe_2_ lattice can capture > 95% of the neutrons in a wafer with a thickness of ~ 3.4 mm^13^. The facile preparation process and high crystal quality of LiInSe_2_ can ensure the high performance of the ^6^LiInSe_2_-based neutron detectors.

Although many studies have been devoted to the optimization of the crystal growth to obtain high-quality crystals, the optical and electrical efficiencies are still inadequate for high-resolution detection due to the presence of defects^14–17^. In general, the optical and electrical behaviors are highly dependent on the crystal defects because the formed defects can distort the energy band structure, decrease the carrier concentration and scatter the charge. Theoretical calculations indicate that defect-free LiInSe_2_ is a yellow crystal with a band gap of ~ 2.86 eV at room temperature^18–21^. As the antisite defects of In-substituted Li (In_Li_^2+^) and Li vacancies (V_Li_^−^) form due to a lack of Li, the strong shift in the absorption edge results in the transformation of the crystal color to red^16^. In addition, crystals containing defects exhibit a decrease in the charge mobility-lifetime product (*μτ*) of the detectors to 3–7.6 × 10^−6^ cm^2^/V due to the charge scattering by the defects^18^. As a result, yellow LiInSe_2_ crystals tend to exhibit better optical and electrical properties.

During LiInSe_2_ synthesis and growth, red crystals with defects are prone to deviation from the stoichiometric ratios due to the loss of elements^10^. Some optimization techniques for yellow LiInSe_2_ crystal growth have been carried out and include annealing, regulation of growth parameters, and stoichiometric ratio modulation; among these, stoichiometric ratio modulation is considered the most effective strategy^22^. In the Siemek study, the color of the LiInSe_2_ crystals varied from green to yellow to pink and further to red during the process of annealing in Se vapor^23^. Vijayakumar attempted to optimize the synthesis process to prepare LiInSe_2_ single crystals by controlling the stoichiometric ratio^24^. However, the proper adjustment of stoichiometry to suppress defects during melt growth remains a major challenge. Therefore, further exploration of the stoichiometric ratios for control of the defects inside the LiInSe_2_ crystals of different colors is necessary for clarifying the structure–performance relationship, optimizing crystal growth, and guiding LiInSe_2_ neutron detector fabrication.

In this study, LiInSe_2_ single crystals with different stoichiometric ratios of Li_0.87_In_0.98_Se_2_, Li_0.94_In_0.99_Se_2_, Li_0.99_In_1.02_Se_2_, and Li_1.01_In_1_Se_2_ were grown by the vertical Bridgman method. A yellow single crystal was obtained from Li_1.01_In_1_Se_2_ when excess Li was used. The band gap of the resulting yellow crystals reached 2.83 eV at room temperature; this value was consistent with the theoretical result of 2.86 eV. In addition, the relationship between the defect concentration inside LiInSe_2_ and the neutron detection performance was studied by photoluminescence spectroscopy. This mechanism analysis for the defect structure impacts of the LiInSe_2_ semiconductor on the neutron detection performance can promote its application in neutron detection.

## Experiment

### Synthesis and crystal growth

LiInSe_2_ was synthesized using pure Li (4N), In (6N), and Se (6N). In this experiment, polycrystalline LiInSe_2_ was directly prepared by a solid-state reaction with high-purity Li, In and Se under a pressure of 5 × 10^−5^ Pa. and 925 °C. To prevent the evaporation of Se and the reaction between Li and the quartz ampoule bottle, both Li and Se were added in excess. During the growth process, the LiInSe_2_ single crystal was grown by the vertical Bridgman method with quartz with a carbon layer and graphite crucibles inside. The polycrystalline material was placed in a growth furnace, and the furnace temperature was increased from room temperature to 950 °C; afterward, the material was soaked at 950 °C for 2 h. The crucible was then moved to the starting position before being controlled to descend at a rate of 0.5 mm/h. A temperature gradient of 10 °C/cm was achieved in the growing area of the furnace. After growth, the LiInSe_2_ crystal was cooled to room temperature. The chemical compositions of the as-grown crystals were quantified using an inductively coupled plasma optical emission spectrometer (ICP‒OES: Optima 8300). The resulting compositions were identified as Li_0.87_In_0.98_Se_2_, Li_0.94_In_0.99_Se_2_, Li_0.99_In_1.02_Se_2_ and Li_1.01_In_1_Se_2_; these were very close to the stoichiometric ratio of LiInSe_2_. The detailed growth parameters of the above crystals are given in Table 1. The uncertainty of element determination in the experiment is less than ± 0.005.

**Table 1:** Synthesis methods for four stoichiometric ratios of different colored crystals.

Sample	Excess Li at.%	Excess Se at.%	Ratio of Li/Se	Crucibles materials	Color
Li_0.87_In_0.98_Se_2_	5	3	1.67	Quartz	Dark red
Li_0.94_In_0.99_Se_2_	5	2	2.5	Quartz	Deep red
Li_0.99_In_1.02_Se_2_	3	2	1.5	Graphite	Light red
Li_1.01_In_1_Se_2_	3	1.5	2	Graphite	Yellow

### Characterization

The transmission spectrum of LiInSe_2_ was obtained by an ultraviolet-vis-near infrared spectrometer (UV‒Vis–NIR spectrometer) and a Fourier transform infrared spectrometer. The band gap of LiInSe_2_ and the defects in the crystal were analyzed by diffuse reflectance spectroscopy and photoluminescence spectroscopy, respectively. The UV‒vis–NIR transmission spectra and diffuse reflectance spectra were recorded in the wavelength range of 200–2200 nm with a UV‒Vis–NIR spectrometer, and BaSO_4_ was used as the 100% reflectance standard. Infrared transmission spectra were recorded in the wavelength range of 2.5–20 μm with a Nicolet Nexus Fourier transform infrared spectrometer. The photoluminescence spectra were measured using a He-Cd laser with an output wavelength of 325 nm and a maximum output power of 25 mW. A TRIAX 550 spectrometer was used to record the photoluminescence (PL) emission with an error range of ± 0.3 nm. A low-temperature sample compartment was used to hold the sample with liquid nitrogen as the freezing medium, and the variable temperature range was 10 K to room temperature.

## Results and discussion

### UV‒vis–NIR transmission and diffuse reflectance measurements

**Figure 1:**
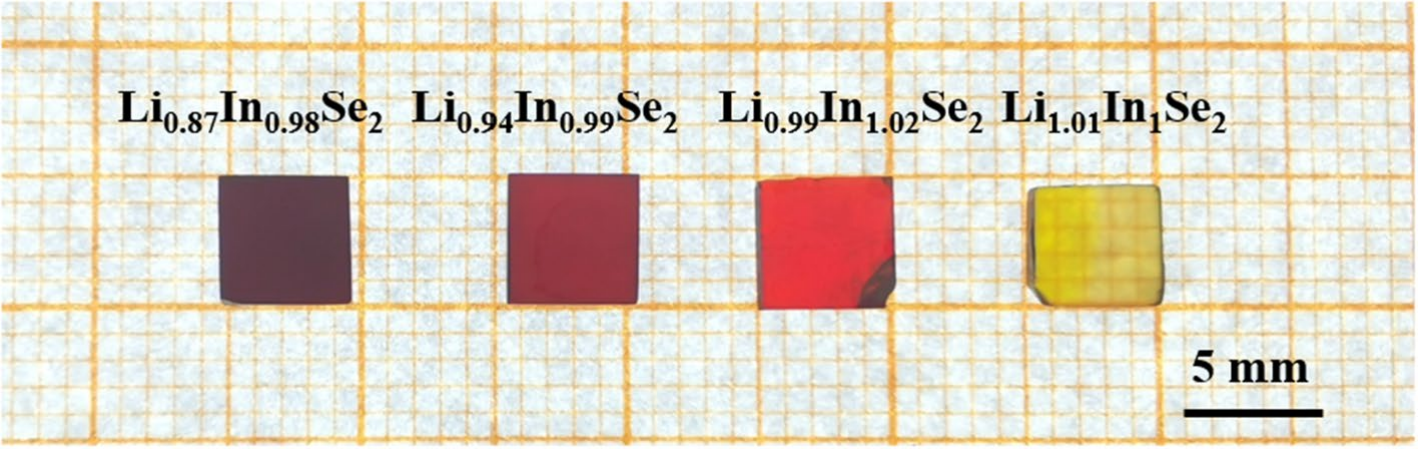
LiInSe_2_ wafers with different color from dark red (Li_0.87_In_0.98_Se_2_), deep red (Li_0.94_In_0.99_Se_2_), light red (Li_0.99_In_1.02_Se_2_) and yellow (Li_1.01_In_1_Se_2_).

**Figure 2:**
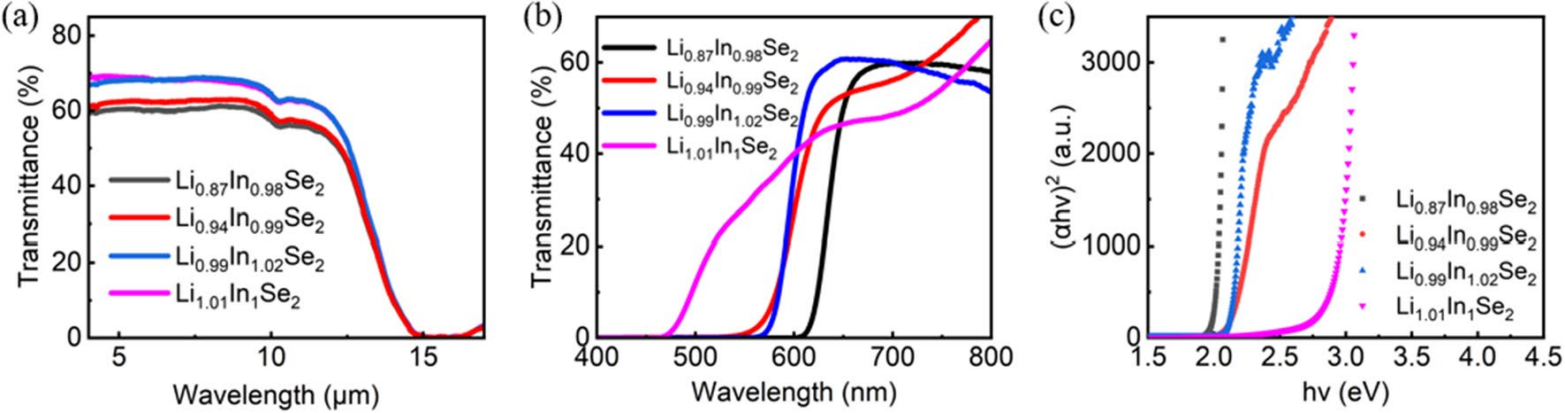
IR (**a**) and UV‒vis–NIR (**b**) transmittance spectra of the LiInSe_2_ wafers with different stoichiometric ratios. (**c**) Tauc plot of (*αhν*)^2^ *vs. hν*.

Figure 1 shows LiInSe_2_ wafers with four different colors: the colors of Li_0.87_In_0.98_Se, Li_0.94_In_0.99_Se, Li_0.99_In_1.02_Se_2_ and Li_1.01_In_1_Se_2_ are dark red, deep red, light red, and yellow, respectively. Specifically, a higher Li content in the crystals contributes to a lighter color. To investigate more clearly the effect of the Li content on the optical properties of LiInSe_2_ wafers, we analyzed the IR and UV‒vis–NIR transmission spectra of the LiInSe_2_ wafers shown in Fig. 2a and b, respectively. The transmittance of the Li_0.94_In_0.99_Se_2_, Li_0.99_In_1.02_Se_2_ and Li_1.01_In_1_Se_2_ crystals reached 70% at wavelengths of 0.8–9 μm, while the Li_0.87_In_0.98_Se_2_ crystal displayed a narrower transmission range and inferior transmission performance. From the transmission spectrum of LiInSe_2_ in the ultraviolet‒visible band (Fig. 2b), the absorption edges of the transmission spectra of the four chips vary considerably in the UV direction, with 610 nm for the Li_0.87_In_0.98_Se_2_ crystal, 538 nm for the Li_0.94_In_0.99_Se_2_ crystal, 565 nm for the Li_0.99_In_1.02_Se_2_ crystal to 436 nm for the Li_1.01_In_1_Se_2_ crystal. Similarly, the resulting optical band gaps of the Li_0.87_In_0.98_Se_2_, Li_0.94_In_0.99_Se_2_, Li_0.99_In_1.02_Se_2_, and Li_1.01_In_1_Se_2_ crystals are ~ 1.98 eV, 2.05 eV, 2.09 eV and 2.83 eV, respectively, by using a Tauc plot (see Fig. 2c)^25^. Importantly, our resulting band gap of ~ 2.83 eV for the Li_1.01_In_1_Se_2_ crystals is highly consistent with the given band gap of ~ 2.86 eV for the theoretical defect-free LiInSe_2_ yellow crystals at room temperature^20–23^. This result confirms that the yellow Li_1.01_In_1_Se_2_ crystals we obtained are high-quality single crystals that closely match to theory.

**Figure 3:**
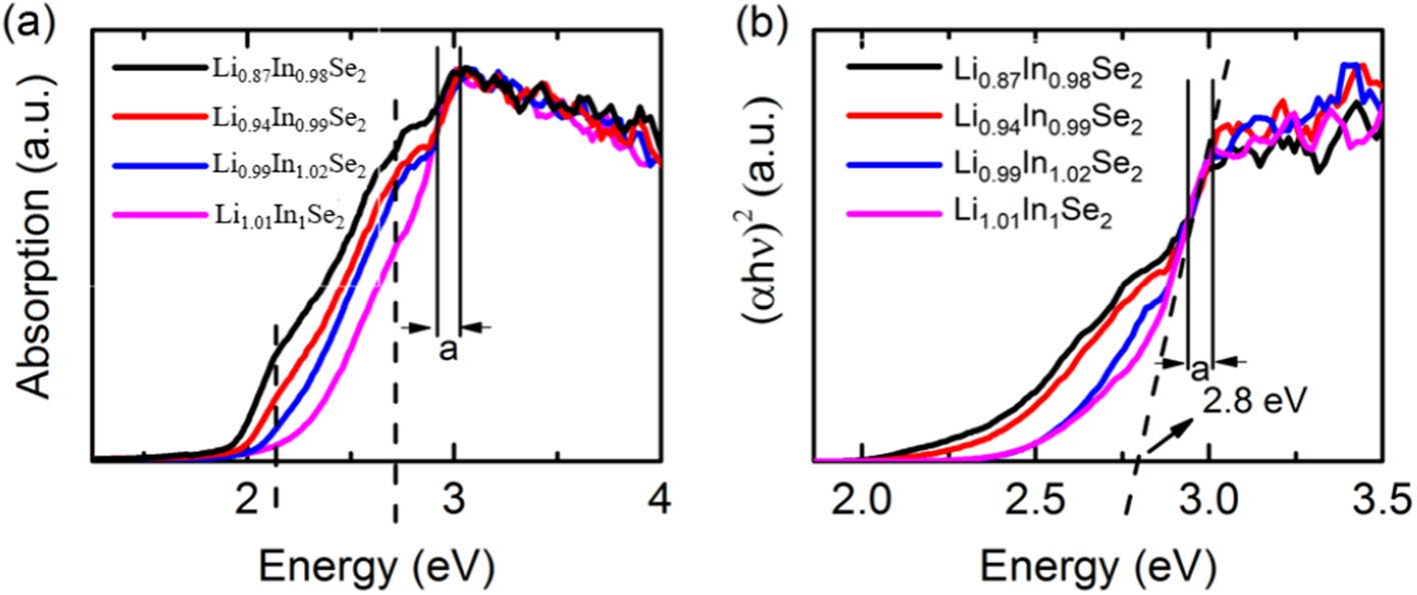
(**a**) Absorption spectra of the as-grown LiInSe_2_ crystals with different stoichiometric ratios and (**b**) Kubelka–Munk plot of (*αhν*)^2^ *vs. hν*.

According to the Kubelka–Munk formula^19–21^, diffuse reflectance spectral data were processed to obtain the powder absorption spectra of the Li_0.87_In_0.98_Se_2_, Li_0.94_In_0.99_Se_2_, Li_0.99_In_1.02_Se_2_ and Li_1.01_In_1_Se_2_ crystals (Fig. 3a). The strongest absorption for all samples occurred in region “a”; this is the real absorption edge of the LiInSe_2_ crystals since the photon energy can be strongly absorbed as it approaches the band gap. Combining the Tauc equation^25^ with absorption spectra, the spectra of (*ahν*)^2^ with respect to *hν* for the Li_0.87_In_0.98_Se_2_, Li_0.94_In_0.99_Se_2_, Li_0.99_In_1.02_Se_2_ and Li_1.01_In_1_Se_2_ crystals are shown in Fig. 3b. The spectral lines of all the samples overlap in region, and the absorption coefficients reaches the maximum values at the high-energy edge of region “a.” Therefore, the straight line in region “a” is the real band gap absorption edge of the LiInSe_2_ crystal. As depicted in Fig. 3b, the intrinsic band gap of LiInSe_2_ can be estimated to be 2.83 eV; this corresponds to the sample Li_1.01_In_1_Se_2_ crystals derived from transmission spectra.

Note that internal defects have a significant influence on the results of transmission spectra as the light passes through the crystal. In contrast, since only light reflected at the crystal surface can be collected in the reflection spectra, the defects within the crystal have a negligible effect on the spectrum. Therefore, the transmittance spectra showing the strong absorption in the red region due to the high concentration of defects inside the crystal likely leads to a redshift of the red crystal.

### Photoluminescence spectra

**Figure 4:**
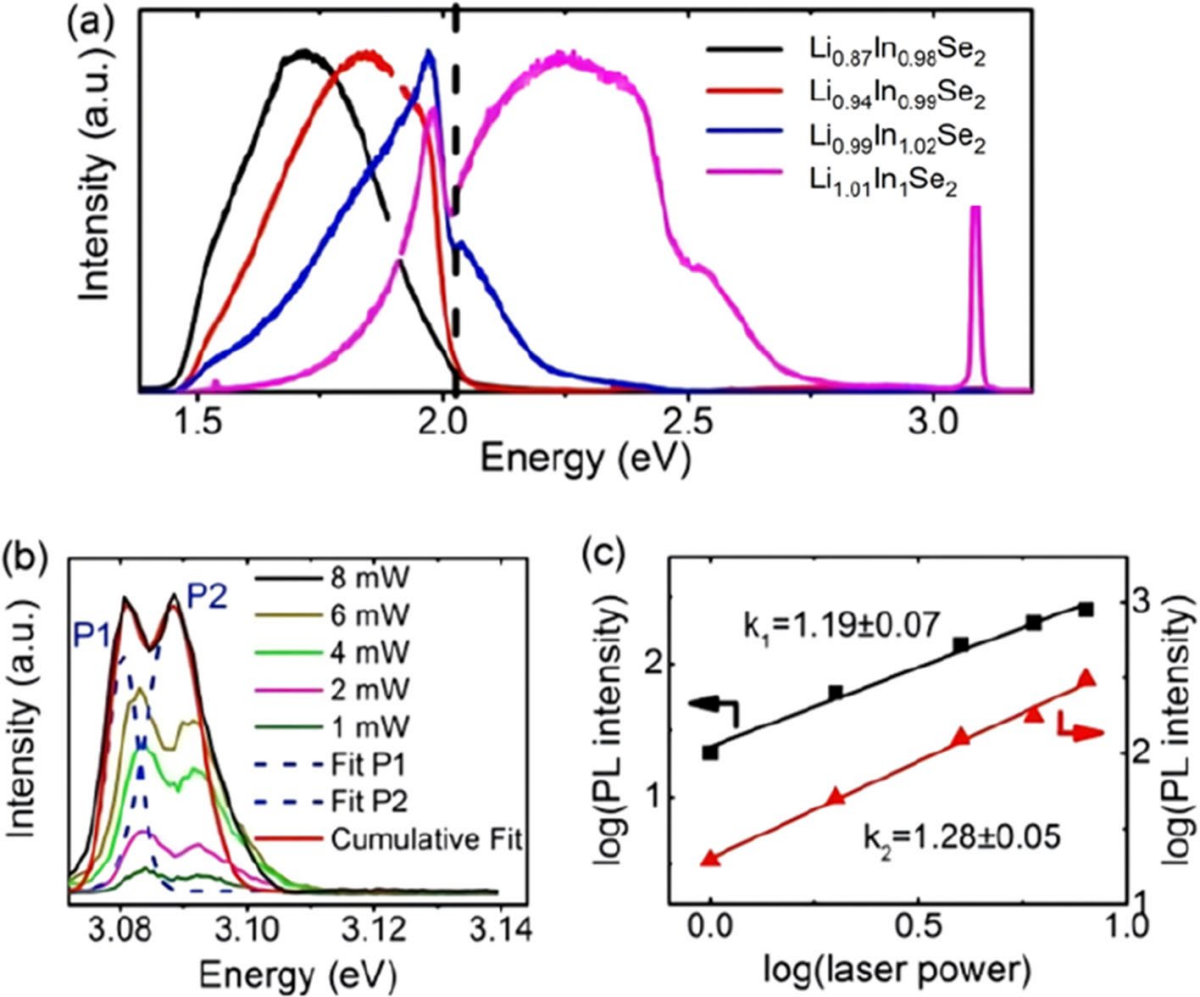
(**a**) PL spectra of LiInSe_2_ wafers with different stoichiometric ratios at 10 K, (**b**) PL spectra of LISe-Y at ~ 3 eV at 10 K under different laser powers, and (**c**) plot of the PL intensity *vs.* laser power.

The PL spectra of the Li_0.87_In_0.98_Se_2_, Li_0.94_In_0.99_Se_2_, Li_0.99_In_1.02_Se_2_ and Li_1.01_In_1_Se_2_ wafers are shown in Fig. 4a. Clearly, the Li_1.01_In_1_Se_2_ crystal shows a wide resulting luminous band in the PL spectrum from 1.5 to 3.1 eV. To analyze the photoluminescence type of the peak at ~ 3 eV, we measured the luminescence of the Li_1.01_In_1_Se_2_ crystal using a laser power ranging from 1 to 8 mW, as shown in Fig. 4b. Gaussian bimodal fitting was carried out for the photoluminescence peaks between 3.07 and 3.11 eV. The Gaussian peak centers of P1 and P2 are 3.08 eV and 3.09 eV, respectively, according to the deconvolution analysis of the PL spectrum (Fig. 4b). The photoluminescence types of the two peaks are determined by the relationship between the laser power and the PL intensity, as follows:

1 $$ I \propto L^{k} $$

where *I* is the PL intensity, *L* is the laser power, and *k* is the index associated with the photoluminescence type. When 0 < *k* < 1, donor–acceptor pair (DAP) emission occurs. When 1 < *k* < 2, free exciton (FE) emission occurs. The resulting *k* values of P1 (*k*_1_) ~ 1.19 and values of P2 (*k*_2_) ~ 1.28 are shown in Fig. 4c, indicating that the two emission peaks P1 and P2 both correspond to FE emission.

The emission peaks in the PL spectra of Li_0.99_In_1.02_Se_2_ to Li_0.87_In_0.98_Se_2_ redshift because of the presence of deeper defects ^[16,26)^. Considering the results in the transmittance spectra, the defect concentrations in Li_0.99_In_1.02_Se_2_, Li_0.94_In_0.99_Se_2_ and Li_0.87_In_0.98_Se_2_ successively increase, and the energy of the excitation peaks in the PL also continuously decreases. Due to the high concentration and diversity of deep defects, peak splitting fitting is difficult to perform, and the specific defect types cannot be confirmed. Thus, the red crystals are likely attributed to the high concentration of defects. High-concentration defects also have a strong secondary absorption effect on the intrinsic FE photoluminescence of LiInSe_2_.

**Figure 5:**
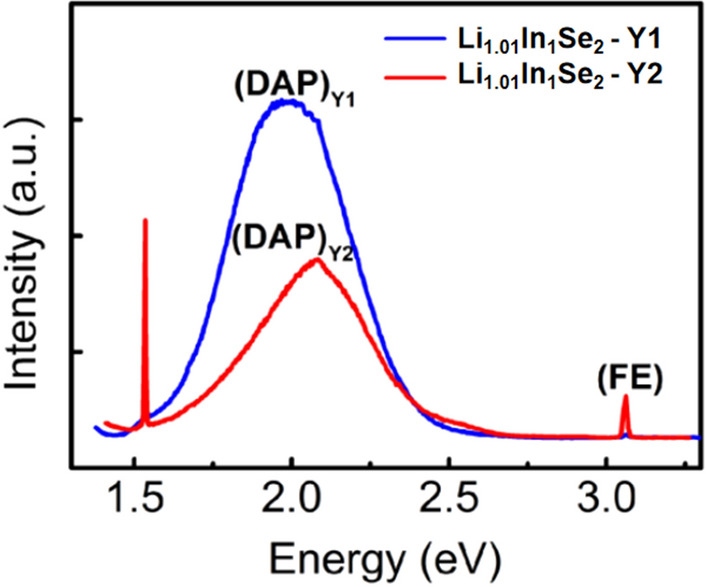
PL spectra of the Y1 and Y2 wafers at 10 K under illumination with a 325 nm laser.

To analyze the possible types of defects inside the LiInSe_2_ crystals, the photoluminescence (PL) spectra of the two yellow wafers, Y1 and Y2, from Li_1.01_In_1_Se_2_ (yellow crystal) are shown in Fig. 5. Both the Y1 and Y2 wafers show DAP emission at 2.08 eV and 1.98 eV and FE emission at 3.06 eV. Specifically, the peak (DAP)_Y1_ conforms to a Gaussian distribution, while (DAP)_Y2_ deviates from a Gaussian distribution.

Considering that E_(DAP)_ = E_g_ − (E_A_ + E_D_) and the defect depth of the resulting defects from Cui et al.^26^, the possible defect types are listed in Table 2.

**Table 2:** Values of the peak wavelength, E_(DAP)_, (E_A_ + E_D_) and corresponding defect type.

Sample	DAP peak	Wavelength	E_(DAP)_	E_A_ + E_D_	Defect type	Ref
Y1	(DAP)_Y2_	595 nm	2.08 eV	0.98 eV	V_Se_^+^ + Li_In_^2−^	This work
Y2	(DAP)_Y3_	625 nm	1.98 eV	1.08 eV	In_Li_^+^ + Li_In_^2−^	This work
Yellow crystal	–	–	–	–	V_Se_^0/+^, In_Li_^0/+^, InLi^+/2+^, V_In_^0/−^, Li_In_^0/−^, Li_In_^−/2−^	^26^
Red crystal	–	–	–	–	In_i_^3+^, V_Li_^−^, In_Li_^2+^	^1,6^
					In_Li_^2+^, V_Li_^−^	^26^

For DAP_(Y1)_ and DAP_(Y2)_, the E_A_ + E_D_ values at 2.08 eV and 1.98 eV are approximately 0.98 eV and 1.08 eV; these values are consistent with the values of E (V_Se_^+^) + E (Li_In_^2−^) and E (In_Li_^+^) + E (Li_In_^2−^), respectively. In addition, the stoichiometric ratio analysis indicates a higher cation content in the yellow crystals, indicating that V_Se_^+^ and Li_In_^2−^ are more likely to form internal defects in the Li_1.01_In_1_Se_2_ crystal. Note that the inclusion of In_Li_^2+^ and V_Li_^−^ defects in the crystal is the main reason for the red color. In our study, we artificially modulate the stoichiometric ratio to manipulate the type of defects. Due to the added excess Li, the In_Li_^2+^ and V_Li_^−^ defects were suppressed, resulting in the formation of the more desirable yellow crystals. This study also provides a research and experience basis for the subsequent yellow crystal growth of ^6^LiInSe_2_.

## Conclusion

In conclusion, we modulate the stoichiometric ratio of LiInSe_2_ to control the color and the defect type of crystals grown via the vertical Bridgman method. The resulting LiInSe_2_ crystals turn yellow as the stoichiometric ratio of Li increases. The band gap of the yellow Li_1.01_In_1_Se_2_ crystal reaches ~ 2.8 eV, as determined by UV‒vis–NIR transmission and diffuse reflectance spectroscopy; this result is consistent with the theoretical value for LiInSe_2_. The PL spectra of Li_1.01_In_1_Se_2_ indicate that V_Se_^+^ and Li_In_^2−^ have internal defects. The modulation of the stoichiometric ratio effectively regulates the types and colors of the defects. Specifically, In_Li_^2+^ + V_Li_^−^ in the red crystals are converted to V_Se_^+^ + Li_In_^2−^ in the yellow crystals due to the addition of excess Li. This study on yellow ^6^LiInSe_2_ crystal growth and defect analysis can effectively promote the practical application of ^6^LiInSe_2_ neutron detectors.

## Data Availability

The datasets generated during and/or analyzed during the current study are available from the corresponding author upon reasonable request.
